# From neglect to necessity: a community case study on the strategic recovery of 57 ventilators during the COVID-19 pandemic

**DOI:** 10.3389/frhs.2026.1761757

**Published:** 2026-06-30

**Authors:** Meshari Rashed Albuqami, Ghadah Sulaiman Alsaleh, Ibrahim Saud Altrair, Marie Hamed Alqahtani, Fahad Mansour Alanazi, Mohmmed Fahad Alqahtani, Faisal Hawas Alattawi, Abdulaziz Abdullah Alshehri, Abdulrahman Bakhat Alzahrani, Abdulrahman Ayed Alruwaili, Reem Abdulrahman Alshehri, Ali Mohmmed Alshehri, Sarah Hassan Alqahtani

**Affiliations:** 1 Assistant Ministry Agency for Maintenance and Facility Operations, General Directorate of Medical Equipment Maintenance, Regional Affairs Department, Ministry of Health, Riyadh, Saudi Arabia; 2 Global Center for Mass Gathering, Ministry of Health, Riyadh, Saudi Arabia; 3 Emergency Medicine Department, Neom Hospital, Tabuk, Saudi Arabia; 4 Engineering Affairs, Ministry of Health, Riyadh, Saudi Arabia; 5 Localization and Human Capital, Ministry of Health, Riyadh, Saudi Arabia; 6 Maintenance and Facility Operation, Ministry of Health, Riyadh, Saudi Arabia; 7 General Directorate of Medical Maintenance, Ministry of Health, Riyadh, Saudi Arabia; 8 Infrastructure, Ministry of Health, Riyadh, Saudi Arabia; 9 Directorate of Equipment and Replacement, Taif Health Cluster, Taif, Saudi Arabia; 10 Planning, Performance and Follow-up, Ministry of Health, Riyadh, Saudi Arabia; 11 Project Management Office for the Deputyship of Maintenance and Facility Operations, Ministry of Health, Riyadh, Saudi Arabia; 12 Environmental Health Facilities, Ministry of Health, Riyadh, Saudi Arabia; 13 Pilgrims Health, Ministry of Health, Riyadh, Saudi Arabia

**Keywords:** COVID19, pandemic, recovery, strategic, ventilator

## Abstract

**Background:**

The COVID-19 pandemic precipitated a critical global shortage of mechanical ventilators, life-saving devices essential for managing patients with severe respiratory complications. The objective of this Community Case Study is to describe the operational process, stakeholder coordination, and outcomes of a national initiative in the Kingdom of Saudi Arabia to rehabilitate decommissioned ventilators, providing a practical model for other health systems facing similar resource constraints during public health emergencies.

**Methods:**

This retrospective case study analyzed a national initiative to rehabilitate decommissioned ventilators during the COVID-19 pandemic. A systematic, multi-phase operation was executed, starting with a comprehensive inventory that identified 183 decommissioned ventilators across the Kingdom's health regions. A detailed technical assessment was subsequently conducted in collaboration with 13 equipment agents. A zero-cost rehabilitation strategy was implemented, whereby functional parts from non-repairable devices were used to restore others. Each device underwent a rigorous two-stage testing and quality assurance protocol before being cleared for clinical use.

**Results:**

From the initial 183 devices, the initiative successfully restored 57 ventilators (31.1%) to full operational status at zero cost, creating an immediate and critical boost to ICU capacity. These devices were deployed to high-need regions, including the Eastern Region (14), Madinah (11), and the Northern Borders (9). The project achieved a calculated cost avoidance of 6,678,116 SAR, equivalent to the price of 57 new ventilators. Furthermore, 80 non-repairable devices were donated to support local industrial manufacturing, fostering national self-sufficiency.

**Conclusion:**

This initiative demonstrates a highly effective model for rapid crisis response and resource optimization in healthcare. By strategically rehabilitating decommissioned medical equipment, the project not only alleviated a critical shortage during a pandemic but also yielded substantial financial savings and advanced strategic goals for local industry and sustainable maintenance, setting a benchmark for innovation in public health logistics.

## A crisis of capacity and a response of resourcefulness

The COVID-19 pandemic constituted a unique global health crisis, placing healthcare systems worldwide under extreme pressure. It exposed critical vulnerabilities in medical supply chains and stretched the capacity of even the most advanced health infrastructures to their limits (1, 2). Poor infrastructure, inadequate financing, and lack of transparency were identified as key.

The importance of flexible and innovative resource management, strengthening operational infrastructure, and enhancing financing and transparency mechanisms was highlighted as essential to supporting healthcare system continuity and improving preparedness during crises (1), factors that led to healthcare system breakdown during this pandemic (3). Crisis management literature emphasizes the importance of resourcefulness, adaptive capacity, and supply chain resilience during emergencies (4). Key drivers of resilient healthcare supply chain preparedness include stakeholder coordination and resource reallocation, which align with the approach taken in this initiative (5). In healthcare, supply chain disruptions during crises require rapid, localized solutions when external procurement is not feasible (6). Immediate action on resource allocation during such pandemics can considerably improve response effectiveness (7).

Similar ventilator shortages were reported globally during the COVID-19 pandemic, with different health systems adopting various procurement and crisis response strategies (8). Countries with a higher number of ventilators per capita experienced significantly lower COVID-19 fatality rates, highlighting the critical role of ventilator availability in pandemic outcomes (9). The principles of the circular economy, including resource efficiency and material reuse, have been identified as key drivers of sustainability, and these same principles underpin the zero-cost parts harvesting strategy described in this case study (10).

During the second and third quarters of the year 2020, the Kingdom of Saudi Arabia, like many nations, faced a critical and escalating shortage of mechanical ventilators (11). This life-saving equipment became the pivotal tool in managing the most severe cases of the virus, which caused acute respiratory distress (12). The widening gap between the number of patients requiring intensive care and the available functional ventilators presented a clear and present danger to public health, threatening to overwhelm ICU capacities (13). This scenario was not unique to the Kingdom, but it demanded a uniquely national solution. Global supply chains were severely disrupted, and international competition for ventilators had driven prices to extremely high levels, making mass procurement a slow and financially impossible task (14, 15). The situation required an immediate, internal, and cost-effective response to strengthen national ability to withstand the crisis and safeguard public health.

Confronted with this urgent need and the prohibitive costs and logistical challenges of international procurement during a global supply chain crisis, the General Administration for Medical Equipment Maintenance, under the Ministry of Health's Auxiliary Agency for Facilities Maintenance and Operation, was tasked with an innovative solution. The directive was clear: increase ventilator capacity for COVID-19 patients without incurring procurement costs. This mandate required the administration to look inwards, fundamentally changing its operational approach from acquiring new resources to unlocking the value of existing ones. This line of inquiry revealed a hidden, unused asset: the stock of decommissioned ventilators. These were devices that had been utilized, stored, and often forgotten in warehouses across the Kingdom, typically perceived as obsolete or beyond economical repair.

The objective of this Community Case Study is to describe the operational process, stakeholder coordination, and outcomes of a national initiative in the Kingdom of Saudi Arabia to rehabilitate decommissioned ventilators, providing a practical model for other health systems facing similar resource constraints during public health emergencies.

## Operational execution: a phased approach to rehabilitation

### Phase 1: the foundational inventory and assessment

The initiative commenced with a critical first step: understanding the full scope of available assets. A specialized team was mobilized to conduct a comprehensive inventory of all decommissioned ventilators, defined as medical devices that had been utilized, stored, and abandoned in warehouses across the Kingdom's regions for various reasons. This initial reconnaissance, completed within a swift five-day period, identified 183 specific ventilators deemed the primary target for the rehabilitation effort. The technical criteria for evaluating ventilators were determined based on emergency and preventive maintenance reports, as well as the latest three models of this type of equipment. The devices were classified according to specific categories, including: device type, model, age, and the availability and quantity of similar devices.

Simultaneously, a broader inventory analysis was conducted to map the entire ecosystem of out-of-service devices. This wider audit, dated May 6, 2020, revealed a total stockpile of 298 ventilators distributed across 13 authorized agents and encompassing 25 different brands. This included major agents like MEDISERV (with brands including BEAR, CAREFUSION, VELLA, VIASYS, and BIRD), ALDALEE ALRAEDA (with brands including HAMILTON, COVIDIEN, BENNETT, ACHIEVA), and SALEHIYA (with brands including PURITAN BENNETT and NEWPORT), among others. This data provided crucial context, revealing the scale of the challenge and the potential for future phases.

Key findings from this audit revealed the scale and nature of the challenge, highlighting three critical areas: inventory concentration, financial burden, and operational status.

- **Inventory Concentration:** The inventory was heavily concentrated with a few agents. The agent SALEHIYA held the largest single inventory with 108 devices, representing a significant portion of the total stockpile.
- **Financial Burden:** The potential financial impact of standard repairs was substantial. The total identified repair cost for the assessed inventory reached 820,000 SAR. This cost was highly concentrated, with the agent ALDALEEL ALRAEDA alone representing 545,200 SAR (66.5%) of the total, followed by GE (184,800 SAR, 22.5%) and ALQASIBI (90,000, SAR 11%).
- Operational Status: The audit provided a clear triage of the devices’ conditions. A significant finding was that 95 devices (31.9%) were immediately classified as non-repairable. Furthermore, while 224 devices (75.2%) had been fully processed, 74 devices (24.8%) remained under evaluation or inspection. This latter group included devices from smaller agents such as AL KHATEEB (10 devices), IBRAHIM ALMANEH (1 device), and DAR EL NAJAT (2 devices), bringing the total number of devices in the inspection phase to 20. These 57 devices were later included as support for healthcare facilities, with maintenance contracts established after the pandemic.

### Phase 2: strategic planning and agent coordination

Following the inventory, the project moved into an intensive 10-day planning and coordination stage. The General Department of Medical Maintenance directed the agents of the identified 183 devices to conduct detailed technical inspections and prepare feasibility reports on the possibility of repair. The explicit goal was to engineer a mechanism for returning devices to service at zero cost. This was achieved through daily meetings where technical and logistical obstacles were identified and resolved in real-time. A unified inventory model was prepared and distributed to all regions to standardize the cataloging and reporting process. Immediate action on resource allocation during pandemics can considerably improve response effectiveness, supporting the real-time coordination approach taken in this initiative (7).

### Phase 3: the execution framework: resource optimization and two-stage testing

The core of the initiative was a resource optimization strategy that transformed the project from a standard repair operation into a zero-cost model. A formal coordination mechanism was established between regional medical maintenance managers and device agents. The central pillar of this strategy was the systematic recovery of functional components from devices deemed beyond repair. Spare parts were harvested from these non-repairable units and used to restore others, creating a self-sustaining parts ecosystem.

Each device designated for potential rehabilitation underwent a rigorous two-stage testing process with continuous monitoring. This ensured that every ventilator returned to service met all safety and performance standards. Once the inventory was completed, comprehensive inspections were conducted by supplier companies to identify faults in each unit, and spare parts were sourced from similar devices to ensure high-quality and sustainable operation for patient care. We managed this initiative in partnership with the authorized agents of these devices, who as technical specialists provided the technical assessment for each unit and ensured compatibility of sourced parts. After repairing faults, simulation tests were conducted according to the manufacturer's recommendations, and the test results were verified to ensure the readiness of the devices**.**

The process involved:

1. Receiving and sorting devices with the appropriate agents.
2. Directing the implementation of detailed technical reports on the extent of repair possible.
3. Executing the two-stage testing and quality assurance protocol.

### Phase 4: results and triage of the 183-device inventory

The intense effort culminated in the complete triage and processing of the initial 183 ventilators. Table 1 presents a detailed breakdown of the triage outcomes by distributor and brand, including the number of devices repaired at no cost, classified as irreparable, and remaining for future cost-based repair.

**Table 1:** Triage outcomes of decommissioned ventilators by distributor and brand.

Distributor	Brand	Number of devices	Repaired at no cost	Irreparable	The remaining cost for the second phase
Mediserv	Bear	15	0	7	8
	Carefusion	10	1	2	7
	Vella	1	0	1	0
	Viasys	10	1	8	1
	Bird	4	0	2	2
Aldaleel Alraeda	Hamilton	53	21	4	28
Salehiya	Covidien	6	0	0	6
	Bennt	17	4	0	13
	Achieva	9	0	6	3
	N-Puritan Bennet	7	0	0	7
	Newport	67	2	41	24
	Mallinckrodt	1	1	0	0
	Tyco Health Care	1	0	1	0
Drager	Drager	64	14	8	42
Ge	Ge	22	13	0	9
Alqasibi	Carefusion (Mobil)	7	0	0	7
Gulf Medical	Maquet	8	0	0	8
Ibrahim Almaneh	Sle 5000	3	0	0	3
Thmar Aljazira	Vital Air	2	0	0	2
Dar El Najat	Bio-Med	2	0	0	2
Dar Elmuidat	Sechrist	1	0	0	1
Al Khateeb	Stephan	10	0	0	10
12	22	320	57	80	183

The results were categorized as follows:

**Category 1:** Successfully Rehabilitated at Zero Cost: 57 Ventilators.
  This was the primary achievement where these devices were fully restored to operational status and immediately deployed to support COVID-19 patients.
**Category 2:** Designated for future cost-based repair: 46 Ventilators.
  These devices were identified as repairable but required specific parts and labor that incurred costs. They were marked for pending budget approval from the relevant authority.
**Category 3:** Donated for National Industrial Development: 80 Ventilators.
  These devices were classified as completely inoperable and not suitable for repair. “Non-repairable” meant the device could not be operated due to the absence of sensitive parts unavailable in the supplier's inventory, or because the device type was unique with no equivalent units available. In a strategic decision, they were donated to the manufacturing platform of the Saudi Industrialists and Exporters Forum. This donation supported the national strategic goal of localizing ventilator manufacturing by providing units for reverse engineering, design study, and parts harvesting. This approach aligns with circular economy principles, where resource efficiency and material reuse contribute to sustainability goals (10).

### Phase 5: regional deployment and agent-specific outcomes

The 57 rehabilitated ventilators were distributed to regions based on need, demonstrating a data-driven deployment. The regional allocation was as follows: the Eastern Region (14 devices), Madinah (11 devices), and the Northern Borders (9 devices). Jazan's allocation was 6 devices, while Asir and Tabuk were each allocated 5 devices. The deployment also included Makkah (4 devices) and Jeddah (3 devices), ensuring strategic coverage across the Kingdom.

Furthermore, a detailed agent-by-agent breakdown of the 183 devices was compiled, showing the specific contribution of each partner to the three categories (repaired at no cost, designated for future cost-based repair, unrepairable/donated) For example, Drager contributed 14 repaired devices, with 8 unrepairable and 42 remaining for future repair. ALDALEEL contributed 21 repaired devices, with 4 unrepairable and 28 remaining. The data for MEDISERV and SALEHIYA was broken down into sub-categories, showing varied outcomes across different lots or models within their inventory.

### Phase 6: recognition and institutionalization of success

The initiative's success was formally communicated to the Innovation Center at the Ministry's Directorate through the “Pioneers Award” program, which aims to support and recognize high-impact staff initiatives. The project received official recognition from His Excellency the Minister of Health, cementing its status as a model of innovation and efficiency within the Ministry.

**Figure 1:**
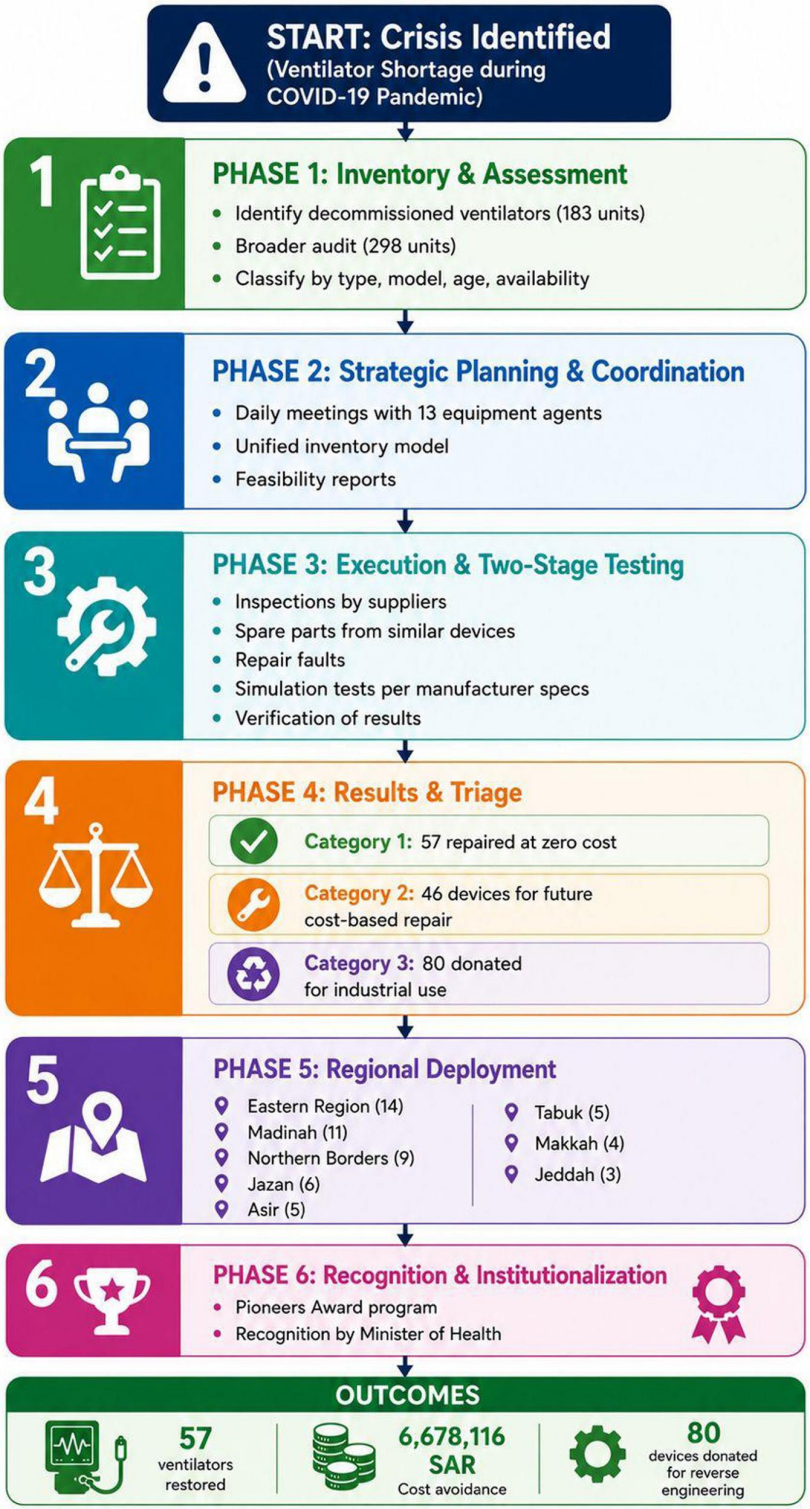
Flowchart of phased rehabilitation process for decommissioned ventilators.

Figure 1 illustrates the complete phased rehabilitation process from crisis identification to final outcomes.

## Outcomes and strategic benefits

The “Rehabilitation and Operation of Decommissioned Ventilators” initiative was successful, delivering substantial impact across operational, financial, and strategic domains.

From an operational perspective, the impact was immediate and critical, as the project provided 57 fully functional, life-saving ventilators to the healthcare system at the peak of a crisis. This initiative did not increase ICU capacity in terms of the number of devices, as these ventilators were already part of the system but had been decommissioned. However, it directly alleviated the critical shortage, boosted ICU capacity, and supported the care of COVID-19 patients across multiple regions.

Furthermore, the financial impact was substantial. By restoring these devices at zero cost, the initiative generated direct cost avoidance. The equivalent purchase price for 57 new ventilators was calculated to be 6,678,116 Saudi Riyals. After completing the repairs, quotations were requested for equivalent ventilator models to calculate the avoided cost of purchasing similar devices. The cost varies depending on the spare parts, as well as the type and model of the ventilator. Labor was provided in cooperation with supplier companies as a national benefit, and logistics services were covered by maintenance contractors at healthcare facilities as in-kind national support. This represents a monumental saving for the Ministry of Health, demonstrating extreme fiscal responsibility and efficiency.

Finally, the initiative yielded a significant strategic impact by achieving several key objectives:

- It supported the Ministry’s second strategic objective of improving the quality and efficiency of health services.
- It established a sustainable model for maintaining costly and essential medical equipment.
- The donation of 80 devices to local industrial platforms supported the Kingdom’s goals for industrial localization and self-sufficiency, turning a liability into a national asset.
- It fostered a culture of innovation, collaboration, and proactive problem-solving within the administration.

This remarkable achievement was not without its significant challenges. The primary challenge was the poor condition of the equipment; these were devices that had been abandoned, with many deemed non-repairable from the outset. A major financial obstacle was the looming repair bill, which was initially estimated at 820,000 Saudi Riyals for the broader inventory. Logistically, the project was immensely complex, requiring the coordination of efforts across the Kingdom's various health regions and with 13 different equipment agents, each with their own processes and parts inventories. To overcome these challenges, the team established a schedule of daily meetings specifically dedicated to presenting and resolving these ongoing obstacles, ensuring that no challenge delayed the project's momentum. Potential errors include the possibility of the devices malfunctioning again due to the inefficiency or unavailability of the required spare parts. Additionally, the findings of this single-site case study may not be directly generalizable to other health systems with different supply chain structures or resource availability.

## Conclusion

In conclusion, this project serves as a notable case study in public sector innovation. This case adds to the literature on health system resilience by providing a practical example of how resource constraints can be addressed through internal asset recovery rather than external procurement. Faced with an urgent crisis, the team avoided conventional solutions and instead unlocked immense value from forgotten resources. Through careful and detailed planning, strategic coordination, and technical excellence, they not only addressed an urgent medical need but also provided a model for resource optimization, contributing to enhanced preparedness and strategic foresight for the Kingdom's healthcare system. This case demonstrates that health system policy should include provisions for internal asset recovery and maintenance capacity during supply chain crises. As such, the phased approach described here, from inventory and triage to parts harvesting and quality assurance, is replicable in other settings facing similar resource constraints during public health emergencies. Stakeholder coordination and resource reallocation are key drivers of resilient healthcare supply chains, which aligns with the multi-agent partnership model used in this initiative (5). Future research could examine the long-term reliability of rehabilitated medical equipment and compare failure rates with new devices. Additionally, studies could investigate the cost-effectiveness of asset recovery models versus traditional procurement in non-pandemic settings. Finally, comparative research across multiple health systems would help identify contextual factors that influence the success of such initiatives. The same asset recovery model could be institutionalized for other critical medical equipment, such as dialysis machines, patient monitors, and anesthesia machines.

## Data Availability

The raw data supporting the conclusions of this article will be made available by the authors, without undue reservation.
